# Critical Review on Anti-Obesity Effects of Anthocyanins Through PI3K/Akt Signaling Pathways

**DOI:** 10.3390/nu17071126

**Published:** 2025-03-24

**Authors:** Nidesha Randeni, Jinhai Luo, Baojun Xu

**Affiliations:** Food Science and Technology Program, Department of Life Sciences, Beijing Normal-Hong Kong Baptist University, Zhuhai 519087, China; nidesha.randeni96@gmail.com (N.R.); luojinhai@uic.edu.cn (J.L.)

**Keywords:** PI3K/Akt signaling pathway, GLUT4, FOXO, GSK3β, mTOR, obesity, metabolic diseases, glucose metabolism

## Abstract

Obesity is a global health crisis and is one of the major reasons for the rising prevalence of metabolic disorders such as type 2 diabetes, cardiovascular diseases, and certain cancers. There has been growing interest in the search for natural molecules with potential anti-obesity effects; among the phytochemicals of interest are anthocyanins, which are flavonoid pigments present in many fruits and vegetables. Anthocyanins influence obesity via several signaling pathways. The PI3K/Akt signaling pathway plays a major role with a focus on downstream targets such as GLUT4, FOXO, GSK3β, and mTOR, which play a central role in the regulation of glucose metabolism, lipid storage, and adipogenesis. The influence of critical factors such as oxidative stress and inflammation also affect the pathophysiology of obesity. However, the studies reviewed have certain limitations, including variations in experimental models, bioavailability challenges, and a lack of extensive clinical validation. While anthocyanin shows tremendous potential, challenges such as poor bioavailability, stability, and regulatory matters must be overcome for successful functional food inclusion of anthocyanins. The future of anthocyanin-derived functional foods lies in their ability to overcome hurdles. Therefore, this review highlights the molecular mechanisms of obesity through the PI3K/Akt signaling pathways and explores how anthocyanins can modulate these signaling pathways to address obesity and related metabolic disorders. It also addresses some ways to solve the challenges, like bioavailability and stability, while emphasizing future possibilities for anthocyanin-based functional foods in obesity management.

## 1. Introduction

Obesity has reached alarming proportions worldwide, becoming one of the most prevalent and critical public health challenges of the 21st century. A body mass index ≥ 30 kg/m^2^ is defined as obesity. According to the World Health Organization (WHO), in 2022, approximately 43% of adults aged 18 years and older were overweight, and 16% were obese globally [1]. Non-communicable diseases like type 2 diabetes (T2D), metabolic syndrome, cardiovascular disease, and metabolic dysfunction-associated fatty liver disease (MAFLD) are among the increasing medical issues that co-occur with obesity [2]. As obesity continues to be a leading cause of preventable morbidity and mortality, effective interventions and therapeutic strategies are urgently needed to mitigate its global impact. Obesity is primarily driven by an imbalance between energy intake and expenditure, where excess energy is stored as fat [3]. In addition to appetite, physical activity, and calorie utilization, the pathophysiology of obesity includes intricate relationships between metabolic, environmental, and genetic factors [4]. Central to these processes are disruptions in the regulation of insulin signaling, adipogenesis, lipid metabolism, and inflammatory responses, all of which contribute to the onset of obesity and its associated complications [5].

Aside from the rising prevalence of obesity, current treatments, including pharmacological interventions, have side effects. The majority of anti-obesity drugs have been associated with side effects that vary from gastrointestinal (e.g., nausea and diarrhea), cardiovascular (e.g., increased heart rate and hypertension), and neurological (e.g., mood alterations and insomnia) [6,7]. These side effects limit their long-term use, patient compliance, and efficacy. Thus, there is an increasing interest in the exploration of natural products as safer, more sustainable alternatives for obesity management.

Recent advancements in understanding the molecular mechanisms underlying obesity have highlighted the potential of dietary bioactive compounds as natural therapeutic agents. Among these, polyphenolic compounds such as anthocyanins, which are predominantly found in fruits and vegetables like blueberries, blackberries, grapes, beetroot, and cherries, have gained attention for their potent antioxidant [8], anti-diabetic [9], anti-inflammatory [10], and anti-obesity [11] properties. Anthocyanins are water-soluble pigments responsible for the red, blue, and purple colors of these fruits and are known for their bioactive effects in modulating various signaling pathways involved in metabolic regulation [12].

One such signaling pathway is the phosphoinositide 3-kinase/protein kinase B (PI3K/Akt) signaling pathway, which is integral to regulating cellular processes such as glucose uptake, protein synthesis, lipid metabolism, and cell survival. PI3K/Akt signaling plays a central role in maintaining energy homeostasis by promoting insulin sensitivity and glucose metabolism while inhibiting excessive fat storage. Dysregulation of PI3K/Akt signaling has been strongly implicated in the development of obesity and insulin resistance. In particular, impairments in PI3K/Akt activity led to reduced glucose uptake, abnormal lipid metabolism, and increased adipocyte differentiation, all of which contributed to the accumulation of excess body fat and the development of obesity [13].

Recent research has shown that anthocyanins can modulate the PI3K/Akt signaling pathway to counteract obesity-related metabolic disturbances. By influencing key downstream targets of PI3K/Akt signaling pathway, such as forkhead box O1 (FOXO1), glycogen synthase kinase 3 β (GSK3β), glucose transporter type 4 (GLUT4), and mechanistic target of rapamycin (mTOR), anthocyanins help restore insulin sensitivity, promote lipolysis, and reduce adipogenesis. Additionally, anthocyanins are known to mitigate oxidative stress and inflammation, which are two critical factors that exacerbate dysfunction of the PI3K/Akt signaling pathway in obesity. Through these mechanisms, anthocyanins may offer a novel and natural approach to managing obesity and its associated metabolic complications [13].

Given the growing burden of obesity on global health systems, the exploration of anthocyanins as a therapeutic agent offers promising prospects. This review examines the molecular mechanisms by which anthocyanins modulate the PI3K/Akt signaling pathway and their impact on obesity. Specifically, we will explore how anthocyanins influence key downstream targets of the PI3K/Akt signaling pathway, including FOXO1, GSK3β, GLUT4, and mTOR, as well as their role in reducing oxidative stress, inflammation, and insulin resistance. In summary, this review will provide a comprehensive understanding of the therapeutic potential of anthocyanins in the prevention and management of obesity, offering insights into their role as natural modulators of the PI3K/Akt signaling pathway.

## 2. Methodology

A comprehensive literature search was conducted using multiple scientific databases, including PubMed, Scopus, Web of Science, and Google Scholar, to identify relevant studies on the anti-obesity effects of anthocyanins through the PI3K/Akt signaling pathway. The search strategy involved using a combination of keywords to refine the selection of articles. The keywords included “anthocyanins and obesity”, “PI3K/Akt signaling in adipogenesis”, “flavonoids in metabolic disorders”, “anthocyanin-rich”, “dietary anthocyanin”, ”cyanidin-3-glucoside”, “anthocyanin supplementation”, ”blueberry extract”, “berry extract”, and “anthocyanin extract”. Additionally, molecular targets and pathways such as “mTOR”, “GLUT4”, “AMPK”, “GSK3”, and “autophagy” were incorporated. To ensure broad coverage, terms like “obesity”, “overweight”, “randomized controlled trial”, “in vitro”, “in vivo”, and “clinical” were included. The inclusion criteria for selecting articles were as follows: (1) studies published in peer-reviewed journals from 2020 to 2025, (2) articles available in English, (3) studies focusing on anthocyanin-mediated regulation of obesity through PI3K/Akt signaling, and (4) only original research articles, reviews, and meta-analyses were included, while case reports, short communications and conference abstracts were excluded. The search process involved an initial screening based on titles and abstracts, followed by a full-text review to ensure relevance. Duplicate records were removed, and studies were further filtered based on the strength of their methodology and reported outcomes. This approach ensured the inclusion of high-quality and up-to-date research to critically evaluate the potential of anthocyanins in modulating obesity through PI3K/Akt signaling.

## 3. Anthocyanins and Their Metabolic Impact

**Figure 1:**
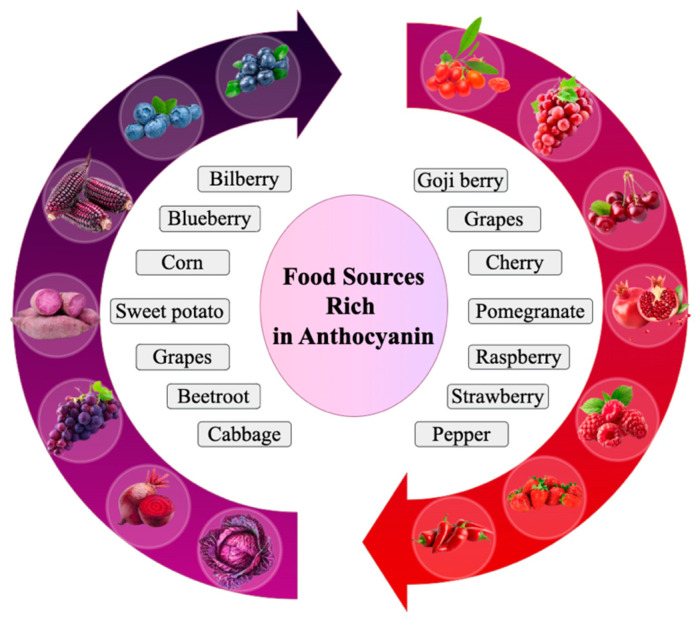
Food sources are rich in anthocyanin. Anthocyanins are natural pigments responsible for the vibrant red, purple, and blue colors found in various plant-based foods. These flavonoids are abundant in berries (e.g., blueberries, bilberries, raspberries, and strawberries), grapes, cherries, pomegranate, goji berries, and vegetables such as purple cabbage, beetroot, and sweet potatoes.

Anthocyanins are a group of water-soluble flavonoids found in most fruits, vegetables, and grains (Figure 1). They are famous for their potent antioxidant [8], anti-diabetic [9], anti-inflammatory [10], and anti-obesity [11] properties. They are responsible for the red, purple, and blue coloration of numerous fruits and vegetables and are generally ingested as a constituent of the human diet, especially in berries like blueberries, strawberries, raspberries, and blackberries, and in other food items like grapes, red cabbage, and eggplant [12].

**Figure 2:**
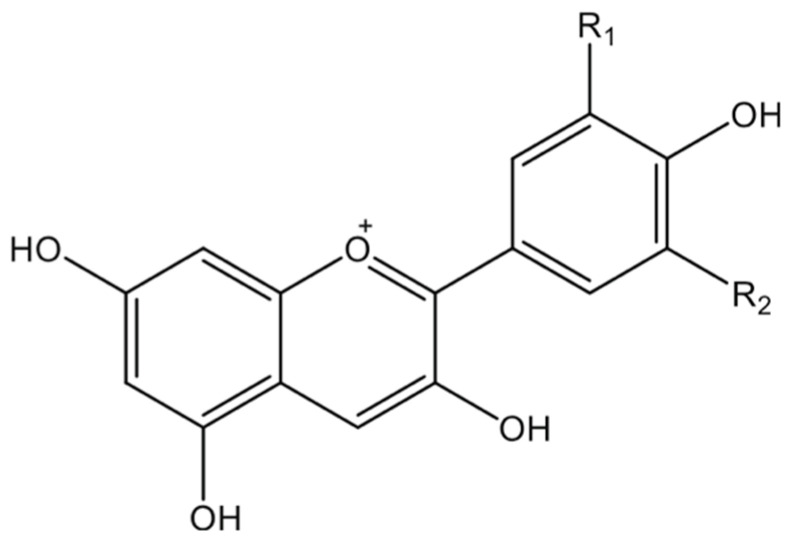
The chemical structure of the anthocyanidin core. The core structure consists of a flavylium cation, which is responsible for its vibrant colors. Hydroxyl (−OH) and other substituents at positions R_1_ and R_2_ determine the specific type of anthocyanidin.

From a molecular standpoint, anthocyanins belong to the subgroup of flavonoids because of their simple carbon chain (C6-C3-C6) structure. Anthocyanin is a molecule that is created when a sugar moiety is joined to the aglycon (anthocyanidin). Anthocyanidin is made up of an aromatic ring joined to another heterocyclic ring that contains oxygen, the latter of which is connected to a third aromatic ring by a carbon–carbon bond (Figure 2). Different functional groups take the place of hydrogen atoms in specific locations on these cyclic structures [14].

Aside from their use as a colorful pigment, anthocyanins have considerable metabolic effects, affecting various biochemical pathways that potentially impact obesity and obesity-associated metabolic disorders. One of the well-documented metabolic effects of anthocyanins is their ability to promote glucose metabolism and insulin sensitivity. Insulin resistance is a feature of obesity and one of the prime movers of T2D pathogenesis. It has been shown that anthocyanins can augment insulin sensitivity through the regulation of diverse signaling pathways, including the PI3K/Akt signaling pathway, which is crucial in glucose uptake by peripheral tissues [15,16,17]. Moreover, obesity is closely linked with dyslipidemia, a state of abnormal blood lipid levels. It includes elevated triglycerides, low high-density lipoprotein, and elevated low-density lipoprotein cholesterol. Dyslipidemia is the cause of atherosclerosis, cardiovascular disease, and fatty liver disease. Anthocyanins have shown significant effects on lipid metabolism, which can reverse the negative lipid profile of obesity [18]. More specifically, anthocyanins were discovered to influence the process of adipogenesis, which is the formation and differentiation of fat cells. In obesity, hyperactive adipogenesis leads to the growth of fat tissue, which is a cause of insulin resistance, chronic inflammation, and metabolic dysfunction. Anthocyanins were discovered to influence the key molecular pathways regulating adipocyte differentiation and lipid accumulation, preventing excessive fat buildup [19].

Chronic low-grade inflammation is a key feature of obesity. It is the primary contributor to insulin resistance, metabolic syndrome, and the development of comorbidities such as T2D and cardiovascular disease. The obese adipose tissue contains infiltrating immune cells, primarily macrophages, which secrete pro-inflammatory cytokines such as tumor necrosis factor (TNF)-α, interleukin (IL)-6, and IL-1β. These inflammatory mediators disrupt insulin signaling and metabolic homeostasis. Anthocyanins possess high anti-inflammatory action, inhibiting inflammatory pathway activation like nuclear factor-kappa B (NF-κB) and mitogen-activated protein kinase (MAPK). By modulating these pathways, anthocyanins restrict pro-inflammatory cytokine biosynthesis and restore insulin sensitivity, which plays a significant role in combating metabolic dysfunction caused by obesity [20].

In addition to their anti-inflammatory effect, anthocyanins are potent antioxidants that inhibit the generation of reactive oxygen species (ROS) and alleviate oxidative stress. Oxidative stress, which occurs when ROS production and antioxidant defense become out of balance, is another cause of insulin resistance and metabolic disease in obesity. Scavenging ROS, anthocyanins maintain cells free from oxidative damage and facilitate the normal functioning of metabolic processes [21].

Recent research has placed the role of gut microbiota in the pathogenesis of obesity and metabolic disease. Dysbiosis, or imbalance of the gut microbiome, is responsible for causing inflammation, insulin resistance, and dyslipidemia. Anthocyanins have been shown to modulate gut microbiota composition [22], promoting a balance between beneficial and pathogenic microbes that influence obesity and metabolic health [23,24]. These compounds can enhance the growth of beneficial bacteria, such as *Lactobacillus* and *Bifidobacterium*, which are known to improve gut barrier function, modulate inflammation, and regulate lipid metabolism [25]. Conversely, anthocyanins can inhibit the growth of pathogenic bacteria such as *Firmicutes* and *Proteobacteria*, which are often associated with dysbiosis, increased gut permeability, and metabolic endotoxemia. A high *Firmicutes*-to-*Bacteroidetes* ratio is commonly linked to obesity, and anthocyanins help restore microbial balance by shifting this ratio in favor of *Bacteroidetes* [26]. Additionally, anthocyanins have been reported to suppress *Escherichia coli* and other pro-inflammatory bacteria, thereby reducing chronic low-grade inflammation, a hallmark of obesity. Through these microbial interactions, anthocyanins contribute to improved gut health, enhanced metabolism, and potential anti-obesity effects. For instance, anthocyanin-rich black corn grain extract has been reported to increase *Bifidobacterium* and *Clostridium* and decrease *E. coli* populations in vivo [27]. Similarly, Wu et al. [28] found that encapsulated and fermented anthocyanin extract significantly modulates the *Firmicutes*-to-*Bacteroidetes* ratio, decreasing the composition and abundance of *Firmicutes* and increasing that of *Bacteroidetes*. These findings underscore the potential of anthocyanins as prebiotic-like compounds that promote a healthier gut microbiome, which may contribute to their anti-obesity effects and overall metabolic benefits.

## 4. Molecular Mechanisms of Anthocyanins in Obesity Through PI3K/Akt Signaling Pathway

**Figure 3:**
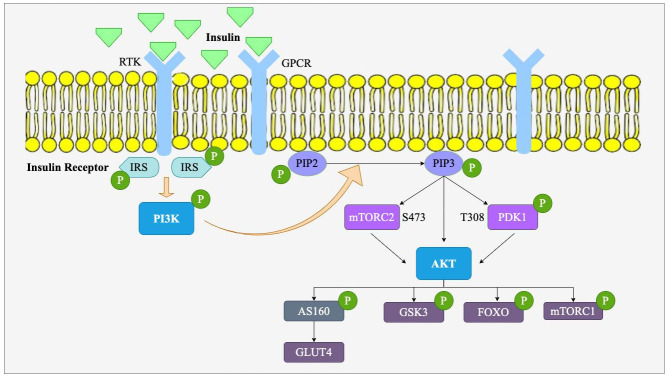
Upstream and downstream targets of the PI3K/Akt signaling pathway. The activation of the PI3K/Akt signaling pathway is initiated by insulin binding to its receptor. Upon activation, insulin receptor substrates (IRS) stimulate PI3K, leading to the conversion of PIP2 to PIP3. PIP3 recruits and activates PDK1 and mTORC2, which phosphorylate Akt at T308 and S473, respectively. Activated Akt regulates multiple downstream targets, including AS160, GSK3, FOXO, and mTORC1.

The PI3K/Akt signaling pathway is among the most significant intracellular signaling pathways involved in the regulation of various physiological processes, including cell survival, proliferation, metabolism, and differentiation [29]. It is stimulated by a wide array of signals, most notably insulin and growth factors, and has a direct impact on metabolic processes. It has a pivotal role in the regulation of glucose metabolism, lipid homeostasis, and insulin sensitivity, which makes it of special interest for the study of obesity and metabolic diseases such as T2D and cardiovascular diseases [13]. As shown in Figure 3, this pathway is triggered when growth factors or insulin are associated with their respective receptors, such as receptor tyrosine kinases (RTK) or G-protein-coupled receptors (GPCR) on the cell surface. RTKs activate insulin receptor substrates (IRS)1/2 through tyrosine phosphorylation, while GPCRs may activate these proteins via serine/threonine phosphorylation or through PI3K activation. IRS1/2 binds to the subunit of phosphoinositide 3-kinase (PI3K) (p85) and leads to the activation of the catalytic subunit of PI3K (p110). This phosphorylation and activation of the enzyme PI3K catalyzes the phosphorylation of phosphatidylinositol-4,5-bisphosphate (PIP2) to phosphatidylinositol-3,4,5-trisphosphate (PIP3). The accumulation of PIP3 in the cell membrane serves as a site of docking for protein kinase B (Akt), followed by phosphorylation and activation by phosphoinositide-dependent kinase 1 (PDK1) at threonine 308 and mTORC2 at serine 473 [13]. Upon activation, Akt regulates a significant number of downstream targets (GLUT4, mTORC1, FOXO, GSK3) that determine essential metabolic events that regulate key cellular functions, including glucose uptake, fat storage, and protein synthesis [30].

In obesity, this pathway is often dysregulated. Obstructive inflammation, oxidative stress, and other aspects of obesity could weaken the activation of the PI3K/Akt signaling pathway to create insulin resistance and visceral fat storage [31]. Insulin resistance, typical in obesity, inhibits proper glucose absorption in tissues and leads to elevated blood glucose levels, a state that contributes extensively to the development of T2D [32]. Furthermore, the impaired Akt function has also been shown to induce abnormal lipid metabolism involving increased fat accumulation in adipocytes and impaired breakdown of fat (lipolysis), which magnifies adiposity and insulin resistance [33]. In Table 1 and Table 2, in vitro and in vivo obesity research targeting the PI3K/Akt signaling pathway through anthocyanins are presented, respectively.

### 4.1. Effect of Anthocyanins on PI3K/Akt/GLUT4 Signaling Pathway in Obesity

In response to the upstream signals, Akt directly regulates GLUT4-containing vesicle translocation to the plasma membrane. Akt phosphorylates and inhibits the Akt substrate of 160 kDa (TBC1D4, AS160), a GTPase-activating protein that maintains GLUT4 vesicles in the cytoplasm. Inhibition of AS160 allows GLUT4 vesicles to translocate to the cell surface, where they fuse with the membrane, allowing glucose to be transported from the blood into the cell. This process enhances insulin sensitivity [13].

GLUT4 is very important in glucose homeostasis. GLUT4 is predominantly expressed in insulin-sensitive tissues such as skeletal muscle, adipose tissue, and cardiac muscle, and its translocation to the plasma membrane is required for glucose uptake. In adipose tissue, GLUT4 facilitates the uptake of glucose into adipocytes and is stored within them as lipids. In muscle tissue, GLUT4 regulates the entry of glucose into the muscle cells to be stored in the form of glycogen or metabolized for energy production [49]. In liver tissue, though GLUT4 has a relatively limited role to play, dysfunction in peripheral tissues indirectly causes dysregulated glucose metabolism, further contributing to insulin resistance and causing MAFLD. In obesity, GLUT4 expression is normally reduced, which results in defective glucose uptake and hyperglycemia. This dysfunction is primarily caused by defective PI3K/Akt signaling, which fails to activate GLUT4 translocation to the cell membrane in response to insulin stimulation [50].

**Figure 4:**
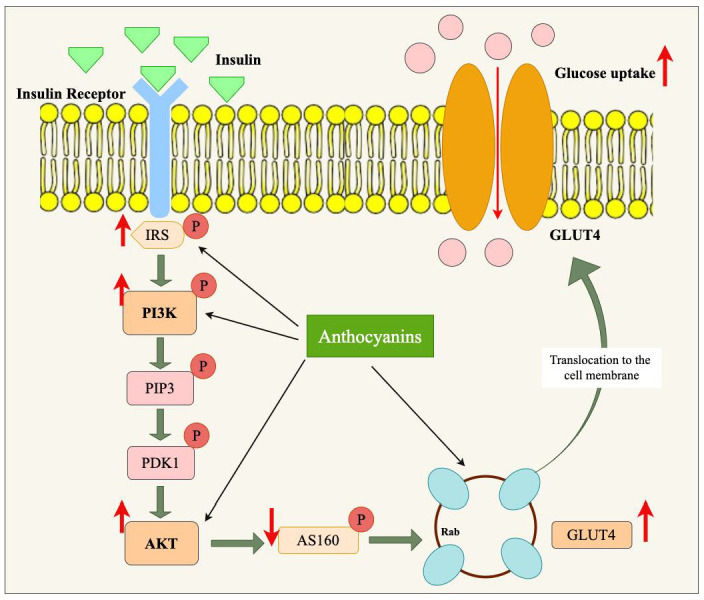
Role of anthocyanin in modulating PI3K/Akt/GLUT4 signaling pathway in obesity. Anthocyanins enhance insulin signaling by upregulating the phosphorylation of key molecules in the PI3K/Akt signaling pathway, including IRS, PI3K, PDK1, and AKT. This leads to the activation of AS160, facilitating the translocation of GLUT4 vesicles to the cell membrane. Consequently, glucose uptake is increased, improving insulin sensitivity and mitigating obesity-related insulin resistance. Upward arrows indicate upregulation, while the downward arrow represents inhibition.

Anthocyanins, by activating the PI3K/Akt signaling pathway, can enhance GLUT4 translocation, improving glucose uptake and helping to restore normal metabolic function in conditions like obesity (Figure 4). Anthocyanins found in blueberries showed an up-regulation of GLUT4 expression extensor digitorum longus (EDL) muscles. In the same study, cyanidin-3-glucoside (C3G) was found to increase the gene expression (PI3K) in EDL. However, neither anthocyanins were sufficient to improve glucose tolerance in obese mice [44]. In an in vitro study by Molonia et al., it was confirmed that C3G anthocyanin showed anti-obesity effects via modulating the PI3K/AKT signaling pathway. C3G restored Akt phosphorylation in cells and restored the gene expression of GLUT-1 and GLUT-4, especially in response to lipotoxicity [20]. Moreover, white sweet potato extracts helped the glucose uptake in TNF-α treated C2C12 cells. The PI3K/Akt signaling pathway mainly mediated the improvement of white sweet potato anthocyanins against TNF-α-induced insulin resistance. This was demonstrated by the IR, IRS-1, and Akt activation by phosphorylation, which might further upregulate GLUT4 expression [16]. Feng et al. found that anthocyanin-rich extracts from black rice could increase glucose uptake in C2C12 myotubes through the promotion of GLUT4 expression in the plasma membrane. In their study, black rice anthocyanins stimulated the phosphorylation of IRS-1 in a dose-dependent manner from 10 to 100 μg/mL while significantly increasing the protein levels of P13K, p-Akt/Akt, and p-p38/p38 [37]. Thus, through the restoration of normal GLUT4 function, anthocyanins increase glucose uptake and reduce blood glucose levels, hence combating insulin resistance and resulting in anti-obesity conditions. Furthermore, anthocyanins have been shown to influence adipocyte differentiation and lipid accumulation, which also indirectly affects GLUT4 expression [45]. By modulating adipogenesis and reducing fat accumulation, anthocyanins help maintain a healthier metabolic profile, promoting better GLUT4-mediated glucose uptake. Thus, anthocyanins not only enhance glucose transport through GLUT4 but also support the broader metabolic processes necessary for maintaining energy balance and combating obesity. Results from an in vivo (HFD/STZ-induced diabetic mice) study show that oral administration (200 mg/kg per body weight (BW)) of purple sweet potato anthocyanins, especially in the group of cyanidin or peonidin-regulated glucose homeostasis by multiple pathways. This was achieved by regulating the expression of proteins (GLUT4) in the glucose transport system, increasing glycolysis, and decreasing gluconeogenesis in the liver [46]. Similarly, in another study, Chen et al. found that anthocyanins from *Aronia melanocarpa* fresh berries had a good potential in regulating glucose uptake. Especially cyanidin-3-O-galactoside, cyanidin-3-O-glucoside, and cyanidin-3-O-arabinoside anthocyanins at the concentration of 40 μg/mL successfully increased the expression of GLUT-4 mRNA and protein levels in both HepG2 cells and C2C12 cells [43]. Moreover, Brightwell rabbiteye blueberries significantly reduced the blood glucose levels from week 2 with continued gradual reduction until week 4 at two doses—100 mg/kg and 400 mg/kg per day, suggesting that dietary inclusion of blueberry anthocyanins for 5 weeks can effectively regulate glucose metabolism and obesity in diabetic mice [38]. Furthermore, Ye et al. stated that in vitro, C3G improved insulin sensitivity through PTP1B and p-IRS-2 modulation. Additionally, it was found that in diabetic db/db mice, C3G intervention improved glucose tolerance and somewhat reduced insulin resistance. Thus, anthocyanin C3G, as a dietary supplement, has the potential to reverse glucose metabolic aberrations [35].

In addition to their direct influence on GLUT4, anthocyanins also control other signaling factors of the PI3K/Akt signaling pathway to further augment the action of insulin. For instance, anthocyanins may reduce oxidative stress and inflammation—both factors implicated in PI3K/Akt impairment under conditions of obesity—through antioxidant and anti-inflammatory activities [21]. These effects contribute to enhanced insulin sensitivity and glucose metabolism, providing hopeful natural therapy for obesity and related metabolic diseases.

### 4.2. Effect of Anthocyanins on PI3K/Akt/FOXO Signaling Pathway in Obesity

FOXO transcription factor family plays a significant role in regulating processes such as cell survival, stress resistance, apoptosis, glucose metabolism, and insulin sensitivity. FOXO proteins are highly sensitive to insulin signaling, and their activity is stringently regulated by the PI3K/Akt signaling pathway. Under normal conditions, FOXO1 is inhibited by the PI3K/Akt signaling pathway, which phosphorylates FOXO1 at conserved threonine and serine residues. This results in preventing its translocation to the nucleus, where it activates gluconeogenic and fat-storing genes. However, in obesity, the impaired PI3K/Akt signaling leads to unphosphorylated FOXO1, which remains localized in the nucleus, promoting the transcription of genes involved in glucose generation (e.g., G6Pase and PEPCK) and lipid storage, leading to hyperglycemia and adiposity [51].

In adipose tissue, FOXO1 activates lipolysis by activating the expression of genes needed for fat degradation, while in obesity, its overactivation can cause an imbalance in the storage and mobilization of fat, which worsens insulin resistance [52]. FOXO1 inhibits skeletal muscle growth and protein synthesis by inhibiting the activity of essential anabolic pathways, such as mTORC1, leading to muscle wasting and impaired glucose metabolism in obesity. In the liver, FOXO1 participates in gluconeogenesis and lipogenesis. This reduces hepatic glucose production, a critical mechanism for maintaining blood glucose homeostasis [51]. These are disturbed by obesity and contribute to hyperglycemia and fatty liver. FOXO1′s role inducing inflammation in several tissues, especially adipose tissue, also adds to chronic low-grade inflammation, an obesity characteristic. In pancreatic β-cells, FOXO1 controls insulin release and β-cell survival, and FOXO1 upregulation is associated with β-cell dysfunction in obesity and the pathogenesis of insulin resistance and T2D. Overall, the disturbed regulation of FOXO factors in obesity results in impaired metabolism, fat accumulation, and increased insulin resistance in organs, reflecting its critical role in obesity-related diseases [13].

**Figure 5:**
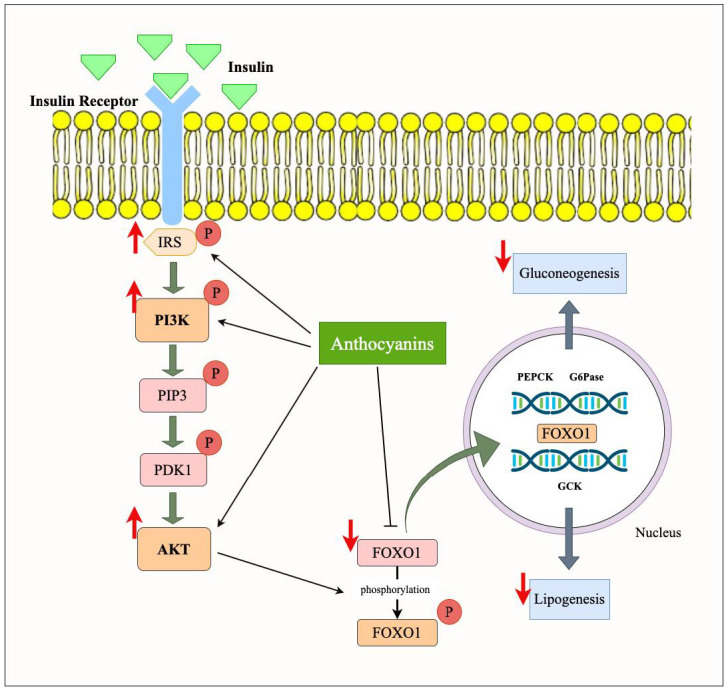
Role of anthocyanin in modulating PI3K/Akt/FOXO pathway in obesity. Anthocyanins enhance insulin signaling by upregulating the phosphorylation of key molecules in the PI3K/Akt pathway, leading to the phosphorylation and inhibition of FOXO1. This prevents FOXO1 from translocating to the nucleus, thereby downregulating the expression of gluconeogenic genes and lipogenesis. These effects contribute to improved glucose metabolism and reduced insulin resistance. Upward arrows indicate upregulation or downregulation of respective pathways.

Anthocyanins, through their ability to activate the PI3K/Akt signaling pathway, help to restore the normal regulation of FOXO1 (Figure 5). More specifically, the anthocyanins promote the phosphorylation and cytoplasmic retention of FOXO1, thereby inhibiting its transcriptional activity. According to a study [44], blueberries decreased the expression of FOXO1 in EDL. Similarly, the mRNA expression of PI3K in the EDL and AMP-activated protein kinase (AMPK) in both muscles was elevated by C3G, a major anthocyanin found in blueberries. According to these findings, blueberries and C3G may affect glucose metabolism by blocking FOXO1 and AMPK/PI3K/AKT/GLUT4 signaling pathways. However, these effects were insufficient to increase glucose tolerance in obese mice. The glucose metabolism regulation by Brightwell rabbiteye blueberries in Herrera-Balandrano et al.’s study was also a result of FOXO1 decrease by anthocyanins [38]. Furthermore, in another study, the expression levels of p-FOXO1/FOXO1 in *Lycium ruthenicum* anthocyanin-treated insulin resistance (IR) Hep-G2 cells were significantly reduced as compared to the normal cells. There are other glucose metabolism routes, such as the c-Jun *N*-terminal kinase pathway, present in IR Hep-G2 cells in addition to the PI3K pathways. Therefore, when IR Hep-G2 cells were pre-treated with the PI3K inhibitor LY294002, the expressions of p-Akt, p-GSK3β, and p-FOXO1 were examined in the same study. Significantly lower levels of activated expressions were observed, indicating that the mechanism by which *Lycium ruthenicum* anthocyanins improved glucose homeostasis was PI3K-dependent [39]. This results in reduced gluconeogenesis, enhanced glucose uptake, and decreased fat storage. By modulating FOXO1, anthocyanins not only improve insulin sensitivity but also mitigate the adverse effects of obesity on glucose and lipid metabolism. Their potential to modulate this key transcription factor highlights the therapeutic promise of anthocyanins in treating obesity-related metabolic disorders.

### 4.3. Effect of Anthocyanins on PI3K/Akt/mTOR Signaling Pathway in Obesity

mTOR is a serine/threonine kinase that regulates critical cellular processes such as growth, protein synthesis, and metabolism. mTOR integrates signals from both nutrients and growth factors and is heavily influenced by PI3K/Akt signaling. Downstream, active Akt phosphorylates and inhibits tuberous sclerosis complex 1/2 (TSC1/2), a GTPase-activating protein that normally inhibits Ras homolog enriched in the brain (Rheb). Repression of TSC2 activates Rheb to bind and phosphorylate mTORC1. Activated mTORC1 upregulates phosphorylation of several downstream substrates implicated in mitochondrial biogenesis, protein synthesis, lipid synthesis, and cell growth. In the context of obesity, the activation of mTOR is often dysregulated, contributing to the accumulation of fat and the development of insulin resistance [53]. In addition to mTORC1, mTORC2 is also activated downstream of PI3K signaling, primarily regulating cytoskeletal organization, cell survival, and metabolic control. mTORC2 activation is influenced by phosphatidic acid and is involved in glucose homeostasis by phosphorylating Akt at Ser473, thereby enhancing insulin signaling. However, in obesity, chronic activation of mTORC2 disrupts insulin signaling, contributing to insulin resistance and metabolic inflexibility [54].

mTORC1 activation in adipose tissue increases the expression of lipogenic genes to promote fatty acid synthesis and triglyceride accumulation, predisposing to excess fat storage and insulin resistance in obesity. In adipose tissue, mTORC1 activation upregulates lipogenic transcription factors, particularly sterol regulatory element-binding proteins (SREBPs), which enhance fatty acid and triglyceride synthesis. This results in increased lipid accumulation, contributing to adipocyte hypertrophy and expansion. Excessive lipid storage predisposes individuals to insulin resistance, as large dysfunctional adipocytes exhibit reduced insulin sensitivity and secrete pro-inflammatory cytokines (e.g., TNF-α, IL-6), further exacerbating metabolic dysregulation. Additionally, mTORC1-mediated inhibition of autophagy in adipose tissue may contribute to the dysfunctional lipid metabolism seen in obesity [55]. In skeletal muscle, mTORC1 regulates protein synthesis and muscle growth via S6K1 and 4E-BP1 activation. However, in obesity, chronic mTORC1 overactivation leads to insulin resistance and muscle wasting by inhibiting IRS-1 phosphorylation and suppressing autophagy, disrupting mitochondrial function [56]. Moreover, mTORC1 influences glycogen synthesis and glucose intake in adipose and muscle tissues by enhancing GLUT4 translocation to the cell surface, thereby increasing blood glucose intake. While this function is beneficial under normal physiological conditions, in obesity, chronic mTORC1 activation leads to insulin desensitization, which paradoxically reduces glucose clearance from the bloodstream. Furthermore, mTORC1 activation in adipose tissue exacerbates lipid accumulation and insulin resistance, creating a vicious cycle that worsens glucose homeostasis in obesity [13]. In the liver, mTORC1 is involved in lipid metabolism, promoting de novo lipogenesis (DNL), the accumulation of excess glucose as fat, which, in obesity, leads to MAFLD and insulin resistance. Further, mTOR is involved in inflammation control, and its activation within liver and fat tissue may enhance chronic low-grade inflammation, a feature of obesity [57]. Additionally, mTORC1 modulates hepatic gluconeogenesis, and its overactivation in obesity may disrupt glucose homeostasis, worsening hyperglycemia, and insulin resistance. Within the pancreas, insulin secretion and β-cell function are regulated by mTORC1, and its pathological regulation results in β-cell dysfunction and insulin resistance during obesity [58]. Persistent activation of mTORC1 can induce endoplasmic reticulum stress and oxidative stress in β-cells, contributing to apoptosis and a decline in β-cell mass [59]. Additionally, mTORC1 overactivity may reduce the expression of key transcription factors involved in insulin gene regulation, leading to decreased insulin synthesis and secretion. Over time, this dysregulation contributes to insulin resistance and hyperglycemia, exacerbating the progression of T2D in obese individuals [60]. Thus, hyperactivation of mTOR in many tissues during obesity causes defective glucose metabolism, increased fat storage, and insulin resistance and is, thus, an important target for the therapy of obesity-associated metabolic disorders. Yet another significant role played by mTORC1 is the inhibition of autophagy, a process by which the cell breaks down and reuses damaged or redundant cellular components. mTORC1 inhibits autophagy by phosphorylating and downregulating the protein Unc-51, Like Autophagy Activating Kinase 1 (ULK1), which is involved in initiating autophagic activities. In periods of nutritional abundance, mTORC1 suppresses autophagy, promoting cell growth and energy storage at its expense [61]. Under low-energy conditions, such as starvation, the AMPK signaling pathway can inhibit mTORC1 activity to conserve energy. AMPK signaling pathway is activated when cellular energy levels are low (i.e., high AMP/ATP ratio), and it phosphorylates and inhibits TSC2, preventing the activation of Rheb and, subsequently, mTORC1. This is an important mechanism for regulating cellular metabolism based on energy availability [62].

**Figure 6:**
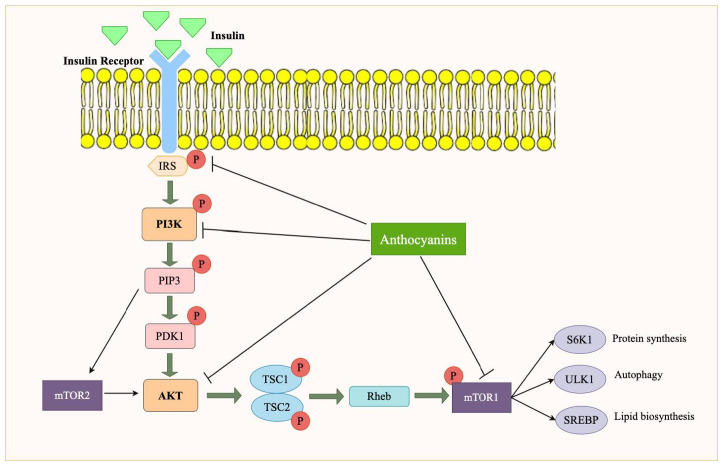
Role of anthocyanin in modulating PI3K/Akt/mTOR signaling pathway in obesity. Anthocyanins influence the PI3K/Akt signaling cascade, leading to the activation of mTORC1 via the phosphorylation of TSC1/TSC2 and Rheb. Activated mTORC1 regulates downstream processes such as protein synthesis (via S6K1), lipid biosynthesis (via SREBP), and autophagy (via ULK1). These effects contribute to metabolic regulation and may help mitigate obesity-related dysfunctions.

Anthocyanins may help regulate mTOR activity, offering a potential mechanism for their anti-obesity effects. By modulating the PI3K/Akt signaling pathway, anthocyanins can suppress excessive mTOR activation, thereby reducing adipogenesis and promoting a healthier metabolic state (Figure 6). Chen et al. used C3G anthocyanin, which is common in fruits and vegetables, for their study with human H1299, A549 cells, and lung epithelial cell line BEAS-2B. At the concentration of 40 μM, C3G lowered the expressions of p-PI3K, p-AKT, and p-mTOR in H1299 and A549 cells by downregulating TP53I3 [41]. Similarly, C3G has shown anti-obesity potential via downregulating phosphorylation of S6K1, which is the substrate of mTORC1. These results were received by Jia et al. in their in vitro study with HepG2 cells and HEK293 cells [36]. In an in vitro study conducted with 4T1 murine BC cells, anthocyanins from Dark sweet cherries downregulated mTOR phosphorylation, showing the potential of anthocyanins to be used as anti-diabetic agents [42]. Additionally, anthocyanins have been shown to modulate autophagy, a process in which damaged cellular components are recycled, which is often impaired in obesity. By restoring mTOR regulation and promoting autophagy, anthocyanins could play a crucial role in reducing the pathological effects of obesity, including excessive fat accumulation and metabolic dysfunction [63]. Moreover, a study on painful diabetic neuropathy found that hyperactivation of the PI3K/Akt/mTOR signaling pathway in diabetic rats suppressed autophagy, leading to increased pain sensitivity. Inhibiting PI3K/Akt/mTOR signaling restored autophagy (Beclin1 and LC3-II expression) and improved pain thresholds, suggesting that mTOR overactivation contributes to metabolic dysfunction in diabetes. These findings reinforce the role of mTOR in metabolic disorders, highlighting its potential as a therapeutic target [64].

### 4.4. Effect of Anthocyanins on PI3K/Akt/ GSK3 Signaling Pathway in Obesity

GSK3 is a serine/threonine kinase that plays a role in the regulation of glycogen metabolism, cell signaling, and insulin responsiveness. GSK3 exists in two forms, GSK3α and GSK3β, and both are involved in various cellular processes, including glucose and lipid metabolism, cell differentiation, and adipogenesis. PI3K/Akt signaling is an important regulator of the activity of GSK3, and Akt-induced phosphorylation inhibits GSK3 with far-reaching consequences on metabolic processes, including glucose homeostasis and fat accumulation. Once Akt is fully activated, it will translocate to the cytoplasm, where it phosphorylates GSK3β at serine 9 (in the case of GSK3β, or serine 21 for GSK3*α*). Phosphorylation at these sites leads to the inactivation of GSK3 by reducing its kinase activity. In its inactive state, GSK3β can no longer phosphorylate and inhibit glycogen synthase, a key enzyme involved in glycogen synthesis. As a result, glycogen synthase remains active and promotes the conversion of glucose to glycogen for storage [65].

GSK3 suppresses lipogenesis through the inhibition of insulin signaling and glucose uptake, leading to dysregulated fat storage and increased insulin resistance in adipose tissue. In skeletal muscle, GSK3 disrupts glucose metabolism through interference with insulin signaling and the inhibition of glycogen synthesis, contributing to muscle insulin resistance and impaired glucose utilization in obesity. In the liver, GSK3 regulates gluconeogenesis and lipogenesis, and its overactivation induces MAFLD and exacerbates hyperglycemia and insulin resistance [66]. Additionally, GSK3’s involvement in inflammation further worsens obesity complications since it promotes the activation of adipose tissue and other organ pro-inflammatory pathways, enhancing the chronic low-grade inflammation of obesity. In the pancreas, GSK3 negatively affects insulin release and β-cell function, resulting in β-cell dysfunction and the development of insulin resistance [67].

Anthocyanins have been shown to modulate PI3K/Akt signaling, leading to the phosphorylation and inactivation of GSK3β. By reducing GSK3β activity, anthocyanins promote glycogen synthesis, enhance glucose uptake, and reduce the accumulation of fat. Li et al. studied the effect of blueberry anthocyanin C3G against high glucose damage in ARPE-19 cells. The results showed that at the concentration of 10 μM, the C3G decreased the protein expression level of GSK3β by promoting phosphorylation of Ser at position 9, especially under the HG-induced oxidative stress conditions in ARPE-19 cells [40]. Furthermore, with *Lycium ruthenicum* anthocyanin, the levels of p-Akt/Akt and p-GSK3β/GSK3β were significantly (*p* < 0.01) reduced as compared to the IR Hep-G2 cell [39]. Herrera-Balandrano et al. conducted an in vitro study with HepG2 cells in order to find out the effects of malvidin-type anthocyanins in Brightwell rabbiteye blueberries. In their study, one of the results was phosphorylation of GSK3β at Ser9 [38]. Similarly, Chen et al. found that anthocyanins from *Aronia melanocarpa* fresh berries, especially cyanidin-3-O-galactoside, cyanidin-3-O-glucoside, and cyanidin-3-O-arabinoside at the concentration of 40 μg/mL successfully increased the IRS-1 levels while reducing levels of GSK-3β expression in both HepG2 cells and C2C12 cells [43]. These findings suggest that GSK3 helps improve insulin sensitivity and alleviates the metabolic disturbances associated with obesity. The ability of anthocyanins to modulate GSK3β activity demonstrates their potential to support glucose and lipid metabolism, making them a valuable therapeutic tool for managing obesity and its related complications.

### 4.5. Cross Link Between PI3K/Akt and AMPK Signaling Pathways

PI3K/Akt and AMPK signaling pathways are both central regulators of energy metabolism but in different contexts. The PI3K/Akt signaling pathway is activated by anabolic stimuli such as insulin release, growth factors (e.g., IGF-1), and nutrient availability. When stimulated, this signaling pathway promotes glucose uptake by increasing GLUT4 translocation, enhances protein synthesis through mTOR activation, and supports lipid storage by inhibiting lipolysis [68]. In contrast, the AMPK signaling pathway acts as an energy sensor and is activated during energy stress conditions such as fasting or exercise. When activated, the AMPK signaling pathway can promote catabolic processes to restore energy balance, including fatty acid oxidation (breaking down fats for energy) and glucose production (gluconeogenesis) while inhibiting anabolic pathways like protein and lipid synthesis [69]. AMPK signaling pathway is activated by an increased AMP/ATP ratio, which is sensed by upstream kinases such as Liver Kinase B1 (LKB1) and Calcium/Calmodulin-dependent kinase kinase 2 (CaMKK2), leading to its phosphorylation and activation. The AMPK signaling pathway regulates cellular glucose uptake by increasing the translocation of glucose transporters GLUT 1 (blood, brain, and kidney) and GLUT 4 (muscle and adipose tissue) [70]. The activation of the AMPK signaling pathway increases the destruction of glucose, fatty acids, cholesterol, and triglycerides in metabolism, preventing their synthesis and storage [71]. By activating autophagy-activating kinase 1/2 (ULK1/2), the AMPK signaling pathway promotes autophagy [71,72]. The AMPK signaling also inhibits mTORC1 activity directly by phosphorylating Raptor and indirectly by activating TSC2, suppressing mTOR signaling, and reducing anabolic processes [60]. It also inhibits protein and rRNA synthesis and phosphorylates several pathway proteins, including mTORC1, FOXO, and EF2, which halts cell growth [9,54,73]. Additionally, the AMPK signaling can promote mitochondrial biogenesis through the activation of PGC-1α, enhancing oxidative metabolism and energy homeostasis [74]. In obesity, AMPK is impaired by excess nutrient availability in the long term [60], leading to decreased oxidation of fatty acids and increased lipogenesis, which increases fat storage and insulin resistance. Chronic nutrient overload also disrupts AMPK-mediated insulin sensitivity by reducing GLUT4 translocation and impairing hepatic glucose homeostasis, further exacerbating metabolic dysfunction [75].

Anthocyanins have been shown to activate both PI3/AKT and AMPK signaling pathways simultaneously, helping to restore balance. For example, anthocyanins can enhance AMPK activity, promoting energy expenditure and lipid oxidation while also improving the PI3K/Akt-mediated insulin signaling, thereby improving glucose uptake and lipid storage in tissues like muscle and adipose tissue. In the study performed by Feng et al., an anthocyanin-rich extract from black rice showed higher glucose uptake potential in the C2C12 cell line. They significantly enhanced the protein levels of p-AMPK/AMPK. Apart from signaling molecules in the PI3K/AKT signaling pathway, these extracts also upregulated GLUT4 glucose uptake by targeting the p38 MAPK/ERK signaling pathway. Thus, anthocyanin-rich extracts from black rice stimulate GLUT4 glucose uptake via the upregulation of PI3K/Akt and AMPK/p38 MAPK signaling in C2C12 myotubes [37]. In an in vivo study by Xu et al., anthocyanins from purple corn blocked the expression of adipogenic transcription factors (C/EBP*α*, ACC, FABP4, and FAS), increased AMPK phosphorylation, and inhibited fatty acid synthase, showing anti-obesity effects [76]. Moreover, anthocyanin extract from maize also showed anti-obesity effects through glucose metabolism in vivo. They successfully activated AMPK signaling, which, in turn, improved insulin sensitivity and protected the body from the overexpression of gluconeogenesis and lipogenesis [77].

### 4.6. Role of Anthocyanins on Oxidative Stress and Inflammation in Obesity Through PI3K/Akt Signaling Pathway

Obesity leads to the overproduction of ROS. The ROS engage in oxidative damage and inflammation, which collectively disrupt key metabolic signaling pathways like the PI3K/Akt signaling pathway. In obesity, ROS can suppress PI3K and Akt activation, leading to insulin resistance, reduction in glucose uptake, and enhancement in fat storage [78].

**Figure 7:**
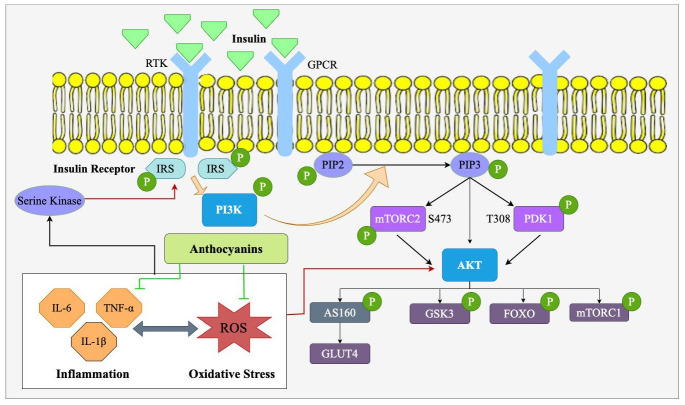
Role of anthocyanin in modulating oxidative stress and inflammation via PI3K/Akt signaling pathway in obesity. Anthocyanins reduce inflammatory cytokines and reactive oxygen species (ROS), thereby decreasing oxidative stress and modulating the PI3K/Akt signaling pathway. This leads to improved insulin signaling, increased glucose uptake via GLUT4 translocation, and better metabolic regulation, contributing to obesity management.

Anthocyanins are powerful antioxidants that help to reverse oxidative stress toxicity effects by eliminating ROS and enhancing the body’s antioxidant status (Figure 7). Anthocyanins have been known to increase antioxidant enzyme activities such as superoxide dismutase (SOD), catalase, and glutathione peroxidase (GPx) that detoxify ROS and stop cellular damage [8]. By reducing ROS levels, anthocyanins restore the oxidant/antioxidant balance that is critical to maintaining the integrity of the PI3K/Akt signaling pathway in obesity [79]. This antioxidant action of anthocyanins is especially essential in adipose tissue, where oxidative stress is typically elevated and causes insulin resistance. Anthocyanins, especially those found in berries like blueberries, have been discovered to possess the potential to enhance PI3K activation and Akt phosphorylation in adipose tissue, muscle, and liver [80]. Restoration of PI3K/Akt signaling through this is pivotal in increasing insulin sensitivity, glucose uptake, and lipid metabolism. In obesity, where oxidative stress typically derails insulin signaling, anthocyanins’ ability to enhance PI3K/Akt activation provides a potential therapeutic intervention for glucose metabolism improvement and insulin resistance relief.

Brightwell rabbiteye blueberry anthocyanin extract showed the potential to reduce ROS levels in vitro [38]. Similarly, C3G in Blueberries also decreased the ROS generation in ARPE-19 cells [40]. Moreover, blueberry anthocyanins have been shown to prevent the activity of antioxidant enzymes from declining with a dosage of 175 mg/kg/BW/day in an in vivo model. The primary constituent of blueberry extracts was mallow anthocyanins, which showed higher antioxidant effects [81]. According to the study by Silveira Rabelo et al., Dark sweet cherry anthocyanins reduced ROS expression in vitro [42]. In another in vitro study by Zhu et al., blueberry anthocyanins showed higher antioxidant activity, especially malvidin-3-O-galactoside, which showed significantly lower scavenging power of both DPPH (38.83) and ABTS+ (14.32) than that of ascorbic acid (112.19 and 44.45, respectively) [17]. Furthermore, anthocyanins from purple sweet potatoes were found to improve the lipid metabolism and activity of antioxidants, reducing tissue damage in HFD/STZ-induced diabetic mice at a concentration of 200 mg/kg (BW) [46]. In another previous study, Black soybean anthocyanin treatment effectively inhibited coculture-induced ROS production. In this study, the ROS level decreased by 40% and 60% with 50 and 100 μg/mL cyanidin-3-glucoside from black soybean, respectively [34].

In addition to their direct antioxidant activity, anthocyanins also reduce inflammation in close correlation with obesity-related oxidative stress. Anthocyanins suppress the inflammatory burden on tissues by reducing effects on oxidative stress and pro-inflammatory cytokine levels [82]. Obesity is a consequence of chronic low-grade inflammation driven by the infiltration of immune cells, such as macrophages, into adipose tissue. These invading immune cells secrete several pro-inflammatory cytokines like TNF-*α*, IL-6, and IL-1β that assist in the development of insulin resistance along with other metabolic dysfunctions [83].

Anthocyanins have been reported to suppress the secretion of these pro-inflammatory cytokines by both macrophages and adipocytes (Figure 7). Suppression of TNF-*α* and IL-6 levels restores a more normal inflammatory response, hence inhibiting the inflammatory condition that induces insulin resistance [84]. In an in vivo study, purple grumixama polyphenols (primarily anthocyanin) ameliorate obesity, insulin sensitivity, and hepatic lipid accumulation, and these effects are associated with the expression of numerous hepatic genes involved primarily in lipid metabolism and inflammation, with an impact on lipid transport, fatty acid transport, and triglyceride hydrolysis [45]. Similarly, treatment with black soybean anthocyanins reduced the migration of macrophages and the generation of inflammatory mediators and cytokines, such as TNF*α*, IL-6, and MCP-1, that were generated by coculture. Black soybean anthocyanin’s restriction of migration may reduce inflammation associated with obesity, given that infiltrating macrophages are a significant source of pro-inflammatory mediators in adipose tissue [33]. Inflammation has been reported to induce the inactivation of the PI3K/Akt signaling pathway, which interferes with metabolic processes [85]. Anthocyanins restore PI3K/Akt signaling pathway function by relieving inflammation-mediated inhibition of the signaling cascade [86]. Restoration increases the sensitivity of insulin, glucose uptake, and fatty acid metabolism in insulin-target organs like adipose tissue, skeletal muscle, and the liver. Thus, anthocyanins can reduce oxidative stress and inflammation and create a favorable environment for the proper functioning of the PI3K/Akt pathway in obesity.

## 5. Clinical Applications

Table 3 provides an overview of several clinical trials examining the effect of anthocyanins on obesity and related metabolic disorders. The trials include different anthocyanin-rich foods such as acai pulp, Medox capsules of blackcurrant and bilberry, jujube berries, strawberry powders, and bilberry juice. The trials were performed to assess different health outcomes, including inflammation, metabolic markers, and body weight in obese or metabolic disorder patients. All of the trials used double-blind, randomized, placebo-controlled designs to the extent possible and had time durations ranging from 6 weeks to 3 months. Inflammation-wise, the majority of the studies emphasized the ability of anthocyanins to reduce pro-inflammatory cytokines such as IFN-*γ*, IL-6, TNF-*α*, and MCP-1 that have a tendency to be elevated in obesity and contribute to insulin resistance and metabolic abnormalities.

For example, Aranha et al. demonstrated that acai pulp supplementation reduced IFN-*γ* and IL-6 levels significantly, resulting in decreased inflammation and improved metabolic outcomes [87]. Their randomized, double-blind, placebo-controlled trial found that consuming açaí pulp for 60 days significantly reduced oxidative stress (*p* = 0.037) and inflammatory markers like IL-6 (*p* = 0.042). The body mass and BMI reduction following acai pulp supplementation show potential anti-obesity effects. However, there were no significant changes in lipid profiles or blood glucose levels, suggesting that while açaí may improve inflammation and oxidative stress, its metabolic benefits remain uncertain. The lack of a long-term follow-up and the relatively small sample size (*n* = 69) may limit the broader applicability of these findings [87]. Medox capsules containing anthocyanins of bilberry and blackcurrant resulted in the reduction of serum levels of IL-6, TNF-*α*, and urinary 8-iso-PGF2*α*, reflecting improvement in oxidative stress and inflammation. Moreover, Basu et al. noted significant decreases in LDL-C levels, increased lipid particle counts, and reduced postprandial PAI-1 levels following strawberry powder supplementation, indicating positive impacts on lipid metabolism and cardiovascular health [88]. The 14-week randomized crossover study demonstrated that a high dose of strawberry powder significantly reduced fasting insulin (*p* = 0.0002) and insulin resistance (*p* = 0.0003). Additionally, reductions in small LDL and VLDL particles (*p* < 0.0001) were observed. However, conventional lipid profiles remained unchanged, and inflammatory markers showed only limited effects, with a significant decrease in serum PAI-1 (*p* = 0.002) [88]. Other studies, for instance, Habanova et al., depicted how bilberry juice led to reduced LDL-C and increased HDL-C, a sign of overall improvement in lipid profiles [89]. Similarly, Jung et al. reported significant reductions in visceral fat, subcutaneous fat, and total fat mass with anthocyanin supplementation, emphasizing the potential role of anthocyanins in obesity and related fat accumulation control [90]. This 12-week double-blind trial in postmenopausal women found that BRE supplementation significantly reduced trunk fat, total fat, and total body fat percentage (*p* = 0.04). However, no significant changes were observed in weight, BMI, or waist circumference. While the study provides preliminary evidence for BRE’s potential anti-obesity effects, the marginal statistical significance (*p* = 0.04) and the absence of metabolic markers such as lipid and glucose levels limit the study’s conclusions [90]. Similarly, a randomized trial demonstrated that 320 mg/day anthocyanin supplementation for 12 weeks significantly improved antioxidant capacity (T-SOD, *p* < 0.05) and reduced inflammatory cytokines such as IL-6 (−40%) and TNF-α (−21%) (*p* < 0.05) [91]. A placebo-controlled trial in obese participants found that juçara pulp supplementation for 6 weeks reduced TLR4 and IL-6 mRNA expression while increasing IL-10 levels. The study provides mechanistic insights into anti-inflammatory effects, but the lack of metabolic outcomes (e.g., lipid profiles and glucose levels) limits its clinical significance. Additionally, the small sample size (*n* = 27) reduces statistical power, necessitating larger trials to confirm these findings [92]. Together, the findings of these studies suggest that anthocyanins may impart a range of metabolic benefits, including anti-inflammatory actions, improvement in lipid metabolism, and aid in weight management, which are of particular significance to obese and obesity-related metabolic disorders.

**Table 3:** Overview of clinical trials examining the effect of anthocyanins on obesity and related metabolic disorders.

Food Matrix	Trial Number	Study Design. Length. Doses	Overweight/Obese Patients: *n*, (Gender), Age	Main Findings	Ref.
Açaí pulp	RBR-72dvqv	Double-blind RPCT 90 days 200 g Açaí pulp. Anthocyanin content was not measured.	69 volunteers	↓Expression of IFN-*γ* and IL-6 ↓Plasma 8-isoprostane concentration levels ↓Body mass and BMI	[87]
Medox capsules; anthocyanins from bilberry and blackcurrant	NCT03415503	Double-blind RPCT 12 weeks 3 doses of anthocyanins supplement (40, 80, and 320 mg/d) 2 Capsules twice a day	169 dyslipidemic participants female and male, 35–70 years	↑Total superoxide dismutase ↓Expression of serum IL-6, TNF-*α* ↓Expression of urinary 8-iso-PGF2α	[91]
Medox capsules; anthocyanins from bilberry and blackcurrant	NCT03415503	Double-blind RPCT 12 weeks 3 doses of anthocyanins (40, 80, and 320 mg/d) 2 Capsules twice a day	176 dyslipidemic subjects female and male, 35–70 years	Significant difference in cholesterol efflux capacity, HDL-C, and apoA1	[93]
Juçara berry (*Euterpe edulis* Mart.)	CEP-UNIFESP No. 0319/2017	Double-blind RPCT 6 weeks 5 g Juçara freeze-dried pulp Anthocyanin content was not measured.	27 obese adult female and male, 31–59 years, BMI: 30.0–39.9 kg/m^2^	↓Expression of TLR4, & IL-6 mRNA ↓Expression of IL-6, TNF-α, and MCP-1	[92]
Black rice anthocyanin	CRIS, KCT0005836	Double-blind RPCT 12-week 500 mg twice daily	105 participants Female 45–69 years BMI ≥ 25 kg/m^2^	↓Visceral fat region, visceral/total (%), and visceral/subcutaneous (%) ↓Trunk fat and total fat mass ↓Body weight and BMI	[90]
Freeze-dried strawberry powders	NCT03441620	Multicenter, double-blind randomized controlled trial 14 weeks 13 g/day and 32 g/day strawberry powder/day	Female and male, BMI ≥ 30 kg/m^2^,	↓Serum LDL-C ↓Postprandial PAI-1 levels	[88]
Freeze-dried strawberry powders	NCT02557334	Double-blinded, RPCT, crossover trial 6 weeks 13 g/d (low-dose), and 40 g/d (high-dose)	Healthy volunteers BMI = 29.4 kg/m^2^ 50 ± 1.0 years	↓TC levels	[94]
Organic bilberry juice	Ethical Committee of the Specialized Hospital of St. Svorad Zobor in Nitra, Slovakia (Study No. 4/071220/202	Single-arm pre-post intervention study 125 mL/day of 100% bilberry juice or 10 g/day of 100% bilberry fiber	30 participants, female, BMI > 30 kg/m^2^, 50–60 years	↓LDL-C and increased HDLC	[89]

Abbreviations: RPCT, randomized placebo-controlled trial; LDL-C, Low-density lipoprotein-cholesterol; HDL-C, High-density lipoprotein-cholesterol; TC, Total cholesterol; ↓, decrease; ↑, increase.

## 6. Anthocyanins in Functional Foods and Pharmacological Synergy for Obesity

The increasing prevalence of obesity and obesity-related metabolic syndromes such as insulin resistance, T2D, and cardiovascular disease have triggered research for new nutritional strategies possessing therapeutic potential for prevention as well as management of these diseases. Functional foods, which are food products with added nutritional value in addition to the minimum amount of essential nutrients, have attracted tremendous attention as a successful way of managing obesity. These foods are typically enriched with bioactive compounds that may affect metabolic processes, increase the sensitivity of insulin, reduce fat storage, and improve overall metabolic wellness [95].

Among these bioactive compounds, anthocyanins have emerged as promising through their anti-inflammatory, antioxidant, and anti-obesity activity. These compounds are naturally found in a wide range of fruits, vegetables, and grains, particularly those with vibrant red, purple, and blue pigments. Berries, such as blueberries, blackberries, and strawberries, are especially rich in anthocyanins, making them excellent candidates for functional food products targeting obesity.

Recent research has also explored the possibility of combining anthocyanin-rich functional foods with conventional anti-obesity drugs to enhance therapeutic outcomes. For instance, studies have suggested that anthocyanins can complement pharmacological treatments by improving glucose uptake, insulin sensitivity, and lipid metabolism while mitigating adverse effects. Additionally, bioavailability remains a key consideration in optimizing anthocyanins’ therapeutic efficacy, as their absorption and metabolism can significantly influence their impact on obesity-related metabolic pathways.

### 6.1. Mechanisms of Anthocyanins in Functional Foods

Anthocyanin-induced activation of the PI3K/Akt signaling pathway can increase insulin sensitivity and glucose uptake in tissues like fat tissue and muscle tissue [80]. They were found to inhibit lipogenesis via the repression of the key lipogenic gene expressions [96]. They augment lipolysis and beta-oxidation of fatty acids, reduce fat storage, and maintain normal body weight [97]. Chronic inflammation is a hallmark of obesity and contributes to insulin resistance, glucose intolerance, and other metabolic dysfunctions. Anthocyanins are effective anti-inflammatory agents with the ability to inhibit the production of pro-inflammatory cytokines such as TNF-*α*, IL-6, and IL-1 [98]. Anthocyanins have been proven to increase insulin sensitivity, maintain a healthy weight balance, and inhibit the onset of obesity-related metabolic disorders by inhibiting inflammation. Oxidative stress is the basis of the pathogenesis of obesity. Anthocyanins, being strong antioxidants, possess the ability to scavenge ROS effectively and guard cells, tissues, and organs against oxidative damage. Anthocyanins inhibit insulin resistance and metabolic disorders by antagonizing oxidative stress [99].

### 6.2. Types of Functional Foods with Anthocyanins

Beverages such as smoothies, juices, and teas are typical delivery vehicles for functional food ingredients since they are easy to consume and are easily absorbed. Anthocyanin-containing berry- and grape-derived beverages can provide an extremely concentrated form of these bioactive substances, sustaining blood glucose equilibrium, reducing inflammation, and promoting weight management [100,101].

Functional foods like dried fruit, granola bars, protein bars, and energy bars are increasingly gaining popularity among health-aware consumers. The incorporation of anthocyanin-containing fruits (like dried blueberries, blackberries, and cherries) and pigmented grains (maize) in these products can provide an easy way to supplement the diet with these compounds. These types of snacks have been found to decrease cravings, balance blood sugar levels, and provide a sustained release of energy without excessive weight gain [102,103,104].

The fortification of common foods with anthocyanins is a powerful way of providing health impacts to large populations of consumers. Breakfast cereals, yogurts, milk products, and bread can be fortified with anthocyanins by incorporating fruit concentrates or extracts. By incorporating anthocyanins in staple foods, food companies can provide consumers with the benefit of improved insulin sensitivity, reduced fat deposition, and improved metabolic health, and also assist in weight management [90,105,106].

For others who need a more intense anthocyanin source, dietary supplements such as powders, capsules, or tablets are an option. Anthocyanin supplements could deliver therapeutic doses of bioactive compounds to support glucose regulation, fat metabolism, and inflammation reduction. These supplements would be particularly beneficial for obesity and metabolic disorder patients who require additional assistance in managing their condition [84,107,108].

Functional oils with anthocyanins from berries or other fruits could be used in salad dressings, cooking oils, or spreads. These oils could combine the antioxidant and anti-inflammatory properties of anthocyanins with the health benefits of healthy fats to enhance both metabolic health and weight management [109,110].

### 6.3. Management of Anthocyanins as Adjuncts to Anti-Obesity Therapies

Anthocyanins have garnered increasing attention for their potential role in obesity management, not only as standalone therapeutic agents but also as complementary compounds alongside existing anti-obesity drugs. Recent advancements in drug development have explored anthocyanin-based formulations and supplements to enhance their bioavailability and metabolic stability. Additionally, emerging research suggests that integrating anthocyanins with conventional anti-obesity drugs may enhance their therapeutic potential while mitigating adverse effects. For example, studies have suggested that anthocyanins can enhance the efficacy of metformin in managing hyperglycemia through multiple mechanisms. Research conducted on insulin-resistant HepG2 cells and a diabetic mouse model demonstrated a significant synergistic effect of anthocyanins and metformin on glucose consumption, with a combination index of less than 0.9, indicating enhanced glucose uptake compared to metformin alone. In vivo, co-administration of anthocyanins (50 and 100 mg/kg) with metformin improved blood glucose levels, reduced insulin resistance, and alleviated organ damage in the liver, pancreas, and ileum. Mechanistically, the combined treatment suppressed protein tyrosine phosphatase 1B (PTP1B) expression and regulated the PI3K/Akt/GSK3β signaling pathway, leading to improved glucose metabolism [111]. Moreover, another study demonstrated that *M. nigra* extract significantly reduced fat content in *C. elegans* in a dose-dependent manner, with a 50.40% reduction at 500 μg/mL and 32.13% at 250 μg/mL. While Orlistat achieved 100% fat reduction, the partial effect of *M. nigra* extract suggests a complementary mechanism, possibly through enhancing lipid metabolism, reducing fat accumulation, or modulating oxidative stress and inflammation. Since anthocyanins have been reported to improve lipid metabolism and insulin sensitivity and reduce oxidative stress, combining them with Orlistat or other anti-obesity drugs could enhance overall weight management outcomes while potentially mitigating side effects associated with pharmaceutical treatments [112]. However, further studies, particularly in mammalian models and human trials, are necessary to confirm the synergistic potential of anthocyanin-rich extracts with anti-obesity and anti-diabetic drugs.

Anthocyanins function as preventive agents, helping to maintain glucose homeostasis, lipid metabolism, and oxidative balance. It helps counteract oxidative stress-induced dysfunction in the PI3K/Akt signaling pathway, which is commonly observed in metabolic disorders. MEDOX^®^ is an anthocyanin-based supplement that has demonstrated potential benefits for individuals with obesity and metabolic concerns. MEDOX^®^, derived from bilberries (*Vaccinium myrtillus*) and blackcurrant (*Ribes nigrum*), was tested in 35 volunteers with normal weight, overweight, and obesity over 29 days at a daily dose of 320 mg of anthocyanins. This supplementation resulted in reduced blood plasma levels of CCL2 cytokine in normal weight, overweight, and obese individuals, as well as a reduction in IL-6 cytokine levels, indicating an anti-inflammatory effect. However, it did not result in significant changes in serum lipid concentrations or anthropometric measurements across the groups. This suggests that while MEDOX^®^ may modulate inflammation, its effects on weight and lipid metabolism may require longer intervention periods or higher doses. These findings suggest that anthocyanin supplementation may support metabolic health by reducing oxidative stress and inflammation, two key factors linked to obesity and related disorders [84]. Understanding these differences is critical in tailoring anthocyanin-based interventions for both prevention and treatment. Future studies should focus on dose-response relationships, bioavailability enhancements, and personalized nutrition strategies to maximize anthocyanins’ benefits across different populations.

### 6.4. Challenges in the Development of Anthocyanin-Based Functional Foods for Obesity Management

#### 6.4.1. Anthocyanin Bioavailability and Stability

The most well-known colored chemicals found in nature are anthocyanins, which are found naturally in fruits, flowers, and vegetables and give them their red, purple, and blue hues. In terms of health, the anthocyanins must be effectively absorbed into the portal circulation and dispersed throughout the body after consumption. However, because anthocyanins are typically ingested in combination with other food sources, the impact of the food matrix on their absorption makes the research of bioavailability more challenging. Numerous investigations have been carried out to comprehend the bioavailability of anthocyanins [113,114]. Emulsions, nanoparticles, drying, and other processes (ionic gelation, centrifugal extrusion, etc.) have all been demonstrated to increase the bioavailability of anthocyanins [115]. Furthermore, anthocyanins are also susceptible to oxidative degradation and environmental conditions such as temperature, light, and pH, which will further limit their stability during the process of food processing, storage, and consumption [116]. Hence, it is a significant challenge to develop functional foods that are capable of maintaining the effectiveness of anthocyanins in the long term.

According to Grgic et al. [117], encapsulation is a technique that encapsulates these compounds in one or more types of polymers to enhance their stability during gastrointestinal digestion and make it easier for the small intestine to absorb prototype molecules. With the help of this technique, anthocyanins can be shielded from harmful environmental elements, including processing, storage, and digestion. They can even be released selectively to improve their health benefits [118]. Various encapsulation techniques have been developed to enhance the stability, bioavailability, and control the release of anthocyanins, ensuring their efficacy in functional foods and therapeutic applications. Polysaccharide-based encapsulation methods utilize carriers such as maltodextrin, cellulose, chitosan and its derivatives, amylopectin, dextran, fructan, and tapioca dextrin, which provide structural integrity and protection against degradation. Protein-based encapsulation, including whey protein isolate and casein, offers improved solubility and controlled release, making anthocyanins more bioaccessible. Lipid-based encapsulation methods, such as liposomes, solid lipid nanoparticles (SLNs), and nanostructured lipid carriers (NLCs), enhance the lipophilicity of anthocyanins, improving their stability and absorption. Additionally, composite material-based encapsulations combine different carrier systems to optimize anthocyanin protection and delivery. These advancements in encapsulation technology play a crucial role in overcoming the limitations associated with anthocyanin degradation and poor bioavailability, thereby enhancing their therapeutic potential in obesity management and metabolic health [119]. For instance, polyethylene glycol and chitosan encapsulation have been shown to preserve both the anti-fatigue benefits and total anthocyanin content for up to one year, even under elevated temperature conditions [120]. Similarly, gelatin/chitosan encapsulation resulted in a retention rate of approximately 50% after 15 days of storage, highlighting its potential to maintain anthocyanin stability over short-term storage [121]. Additionally, a chondroitin sulfate-based nanocomplex with kappa-carrageenan effectively protected anthocyanins during storage by improving their stability across pH 1–6 and at various temperatures (4 °C, 25 °C, 37 °C, and 55 °C) [122]. Moreover, α-casein and β-casein have been reported to improve the stability of anthocyanin monomers while also reducing the loss of antioxidant capacity during in vitro digestion [123]. Furthermore, the consumption of α-casein alongside anthocyanins led to an increase in the maximal concentrations of anthocyanins and their metabolites in rat plasma, suggesting enhanced absorption and bioavailability [124]. Additionally, whey protein isolate has been found to improve anthocyanin stability under light irradiation and inhibit degradation during in vitro digestion, further supporting its role in protecting anthocyanins and ensuring their efficacy in functional food applications [125]. Encapsulation technology, such as nanoencapsulation or liposomal delivery, shows promise, but the feasibility and effectiveness of bulk food production can be explored further.

Anthocyanin absorption varies significantly based on dietary sources and individual metabolic differences. The sugar moiety attached to anthocyanins influences bioavailability. Anthocyanin glycosides are rapidly and efficiently absorbed from the small intestine, whereas their aglycones are less efficiently absorbed [126]. Additionally, dietary components such as fiber, fats, and proteins can modulate anthocyanin uptake [127]. Individual factors, including gut microbiota composition, genetic variations in metabolic enzymes, and physiological conditions like obesity and diabetes, further impact anthocyanin metabolism and excretion [128,129]. Moreover, anthocyanins have low systemic bioavailability due to rapid metabolism and elimination, necessitating strategies like encapsulation to enhance their stability and absorption. Understanding these variations is crucial for optimizing anthocyanin-based dietary interventions and therapeutic applications.

#### 6.4.2. Variability in Anthocyanin Content

Variability in the concentration of anthocyanin in different sources and batches is another challenge. Anthocyanins are present in a variety of fruits, vegetables, and grains, but their concentrations are likely to differ based on plant species, cultivar, environmental conditions, and harvest time. Such variability makes it challenging to ensure a uniform dose of anthocyanins in functional foods [130,131].

To solve this issue, there is a need for standardized extraction processes that yield a relatively uniform number of anthocyanins, along with the development of high-yielding plant varieties with improved breeding for maximum anthocyanin content. In the absence of standardization, it is hard to ensure the delivery of the desired health effects and therapeutic actions by functional foods, particularly in clinical applications.

Other than this, imposing proper regulatory and safety guidelines and addressing overconsumption and misuse conditions need to be considered. This should also be aligned with long-term studies assessing the effects of anthocyanins on obesity and metabolic health.

## 7. Future Directions

The growing need for anthocyanins as bioactive compounds for the treatment of obesity has opened up many paths for their development in functional foods. While promising trends have already begun to materialize, many directions can be pursued in future research to maximize the efficacy, bioavailability, and practical application of anthocyanins in the treatment of obesity and associated metabolic disorders.

One of the main hindrances to the incorporation of anthocyanins into functional foods is that they are poorly bioavailable [132]. In order to overcome this limitation, future research should focus on the development of new delivery systems to increase the bioavailability of anthocyanins. Techniques such as nanoencapsulation [133], liposomal delivery [134], microencapsulation [135], and more could be explored for stabilizing anthocyanins against degradation during digestion and enhancing their delivery into the bloodstream. This would increase the bioactive functionality of anthocyanins upon consumption through food products with function.

While anthocyanins occur in several fruits and vegetables, the content of these phytochemicals can vary significantly depending on the source, cultivar, and environmental factors [136]. To formulate functional foods, it is necessary to have a standardized and consistent anthocyanin content to achieve the desired health benefits. Future studies can be focused on the determination of specific varieties of anthocyanin-containing fruits and vegetables that yield high concentrations of these bioactive constituents. In addition, the production of standardized anthocyanin extracts can make it easier to introduce anthocyanins into a wide range of food products without losing guaranteed potency.

Future studies may explore the synergistic effect of anthocyanins when combined with other compounds such as polyphenols, omega-3 fatty acids, and dietary fiber. Understanding how anthocyanins interact with other nutrients or bioactive compounds can lead to the development of multifunctional foods aimed at addressing multiple aspects of obesity and metabolic derangement.

While preclinical studies and animal models have validated the beneficial effect of anthocyanins on obesity and metabolic syndrome, human clinical trials need to be conducted to validate these findings in clinical situations. Long-term studies with follow-up on the effects of diets containing anthocyanins on weight management, insulin sensitivity, inflammation, and lipid metabolism are needed to prove the effectiveness of these molecules in obese individuals. In addition, clinical trials are required to examine the most effective dosage and how long it takes to gain significant health benefits, so anthocyanin-enriched functional foods might be an actual long-term obesity prevention strategy.

For anthocyanins to be successfully incorporated into functional foods, they would need to undergo rigorous regulatory approval to ensure their safety and efficacy. Safety profiles of anthocyanin-fortified foods and their long-term effects will need to be studied for regulatory approval by health agencies like the FDA, EFSA, and other global agencies. One critical factor that will determine the widespread use of anthocyanin-enriched functional foods will be consumer acceptance of such foods. Future research could aim at consumer attitudes and sensory preferences of foods fortified with anthocyanins to align more with market demand and improve consumer intake.

Food processing technologies that can preserve the stability and bioactivity of anthocyanins will make or break functional foods. Future research could seek to explore less harsh extraction methods (e.g., cold-pressing, freeze-drying, or supercritical fluid extraction) capable of preserving anthocyanin stability during processing. Furthermore, emergent technology like 3D food printing or customized nutrition platforms may be able to deliver anthocyanins with high specificity in specially designed food products tailored to specific metabolic needs.

## 8. Conclusions

Anthocyanins, as natural bioactive compounds, hold significant promise in managing obesity and related metabolic disorders through their modulation of the PI3K/Akt signaling pathway and its downstream targets, such as GLUT4, FOXO, GSK3β, and mTOR. Their ability to influence key factors like oxidative stress, inflammation, and AMPK signaling further underscores their potential therapeutic benefits. Despite challenges related to bioavailability, stability, and regulatory concerns, continued research into improving these aspects, along with clinical validation, could pave the way for the successful development of anthocyanin-enriched functional foods. These advancements could offer a novel, sustainable approach to obesity prevention and management, contributing to the growing field of natural-based therapeutics for metabolic health.

## Figures and Tables

**Table 1:** In vitro obesity research targeting PI3K/Akt signaling pathway through anthocyanins.

Compound	Source	Study Model	Dose	Effects	Reference
C3G		SGBS cells 3T3-L1 cells	10–20 μm	↑Expression of GLUT-1 and GLUT-4 protein levels ↑IRS-1 phosphorylation Glucose uptake ↓Lipid accumulation,	[15]
Petunidin-3-*O*-glucoside, delphinidin-3-*O*-glucoside Malvidin-3-*O*-galactoside	Blueberry	HepG2 cells	100 μm	↑Glucose uptake ↓Lipid accumulation	[17]
C3G	Black soybean	3T3-L1 cells	12.5 μg/mL	↑Expression of pparγ protein ↓ROS levels	[34]
	White sweet potato	C2C12 cells	12.5–200 µg/mL	↑Glucose uptake ↑PI3K/Akt signaling pathway activation	[16]
C3G	Red bayberry	HepG2 cells L02 cells	20, 40, and 80 μg/mL	↑Glucose consumption ↑Glucose uptake	[35]
C3G	Red bayberry	HepG2 cells HEK293 cells	10 and 50 μm	↓Transactivation of FOXO1 ↓Phosphorylation of S6K1 (mtorc1 substrate)	[36]
	Hualien black rice	C2C12 cells	10, 50, and 100 μg/mL	↑Expression of GLUT4 level ↑Phosphorylation of IRS-1 ↑Expression levels of P13K, p-Akt/Akt, and p-p38/p38	[37]
Malvidin	Brightwell rabbiteye blueberries	HepG2 cells	5 μg/mL	↓ROS levels ↓Expression levels of FOXO1 ↑Phosphorylation GSK3β at Ser9 ↓Expression levels of SREBP-1c	[38]
Petunidin-3-*O*-glucoside-5-*O*-galactoside	*Lycium ruthenicum* Murray	Caco-2 cells HepG2 cells	25, 50, 100 and 200 μg/mL	↑Glucose consumption ↑Glucose uptake ↑Protein levels of p-Akt/Akt ↓Activity of the downstream GSK3β and FOXO1 ↑Protein levels of p-IRS2/IRS2	[39]
C3G	Blueberry	ARPE-19 cells	10 μm	↓ROS generation ↓Expression level of GSK3β	[40]
C3G	Fruits and vegetables	Human H1299 and A549 cells Lung epithelial cell line BEAS-2B	40 μm	↓Expression level of p-mtor/ mtor ↓Expression level of p-Akt/Akt	[41]
	Dark sweet cherry	4T1 murine BC cells	-	↓Expression level of p-mtor/mtor ↓ROS levels	[42]
C3G Cyanidin-3-*O*-galactoside Cyanidin-3-*O*-arabinoside	*Aronia melanocarpa*	HepG2 cells C2C12 cells	40 μg/mL	↑mRNA and protein levels of GLUT-4 ↑Glucose uptake ↓Expression levels of p-GSK-3β (Ser^9^)	[43]

Abbreviation; C3G, Cyanidin-3-glucoside; ↑, increase/upregulate; ↓, decrease/downregulate.

**Table 2:** In vivo obesity research targeting PI3K/Akt signaling pathway through anthocyanins.

Compound	Source	Model	Dose	Effects	Reference
C3G	Blueberry	Male C57BL/6 mice, 6-week-old, *n* = 10	Blueberries—6.4 g/kg BW/d C3G—0.02 g/kg BW/d	↓FoxO1 expression in the EDL ↑GLUT4 expression ↑AMPK and PI3K expression in the EDL skeletal	[44]
C3G	Purple grumixama	Male C57BL/6J mice, 7-week old, *n* = 12	200 mg/kg/BW/d	↑Insulin sensitivity ↓Insulin resistance ↓Triglyceride accumulation in the liver ↓Akt1 and Slc1a2 gene expression	[45]
C3G	Red bayberry	Male C57BL/6J mice, 4-week-old, *n* = 6	150 mg/kg/BW/d	↓Blood glucose levels	[35]
cyanidin or peonidin	Purple sweet potatoes	Male Institute of Cancer Research (ICR) mice, 4-week, old, *n* = 6	200 mg/kg/BW/d	↑Glucose tolerance ↑Hypoglycemic activity ↑Glycolysis ↓Gluconeogenesis in the liver	[46]
Pelargonidin-3-*O*-glucoside	Wild raspberry	The (db/db) mice with C57BL/6J background, 6-week-old, *n* = 12	50 mg/kg/BW and 150 mg/kg/BW	↑Glucose tolerance ↑Insulin sensitivity ↓Hepatic genes sterol regulatory element-binding transcription factor 1 (Srebp1c)	[47]
malvidin	Brightwell rabbit eye blueberries	Male C57BL/6J mice	100 mg/kg/BW, and 400 mg/kg/BW	↓Blood glucose levels	[38]
-	Red raspberry	Male C57BL/6J mice, *n* = 12	150 mg/kg/BW	↑Lipolysis ↓Levels of total cholesterol, LDL cholesterol	[48]

Abbreviation; C3G, cyanidin-3-glucoside; BW/d, body weight/day; ↑, increase/upregulate; ↓, decrease/downregulate.

## Abbreviations

The following abbreviations are used in this manuscript:

AMPK	AMP-activated protein kinase
Akt	Protein kinase B
BW	Body weight
C3G	Cyanidin-3-glucoside
DNL	De novo lipogenesis
EDL	Extensor digitorum longus
FOXO1	Forkhead box O1
GLUT4	Glucose transporter type 4
GPCR	G-protein-coupled receptors
GPx	Glutathione peroxidase
GSK3	Glycogen Synthase Kinase 3
IL	Interleukin
IR	Insulin resistance
IRS	Insulin receptor substrates
MAPK	Mitogen-activated protein kinase
MAFLD	Metabolic dysfunction-associated fatty liver disease
mTOR	Mechanistic target of rapamycin
NF-κB	Nuclear factor-kappa B
PDK1	Phosphoinositide-dependent kinase 1
PI3K	Phosphoinositide 3-kinase
PI3K/Akt	Phosphoinositide 3-kinase/protein kinase B
PIP2	Phosphatidylinositol-4,5-bisphosphate
PIP3	Phosphatidylinositol-3,4,5-trisphosphate
Rheb	Ras homolog enriched in the brain
ROS	Reactive oxygen species
RTK	Receptor tyrosine kinases
SGBS	Human Simpson–Golabi–Behmel syndrome
SOD	Superoxide dismutase
T2D	Type 2 diabetes
TNF	Tumor necrosis factor
TSC1/2	Tuberous sclerosis complex 1/2
ULK1	Unc-51 Like Autophagy Activating Kinase 1
WHO	World Health Organization
